# All eyes on the patient: the influence of oncologists’ nonverbal communication on breast cancer patients’ trust

**DOI:** 10.1007/s10549-015-3486-0

**Published:** 2015-07-31

**Authors:** Marij A. Hillen, Hanneke C. J. M. de Haes, Geertjan van Tienhoven, Nina Bijker, Hanneke W. M. van Laarhoven, Daniëlle M. Vermeulen, Ellen M. A. Smets

**Affiliations:** 1 Department of Medical Psychology, Academic Medical Center, University of Amsterdam, P.O. Box 22700, 1100 DE Amsterdam, The Netherlands; 2 Department of Radiotherapy, Academic Medical Center, University of Amsterdam, P.O. Box 22700, 1100 DE Amsterdam, The Netherlands; 3 Department of Medical Oncology, Academic Medical Center, University of Amsterdam, P.O. Box 22700, 1100 DE Amsterdam, The Netherlands

**Keywords:** Physician–patient relations, Trust, Communication, Nonverbal, Video vignettes, Oncology

## Abstract

Trust in the oncologist is crucial for breast cancer patients. It reduces worry, enhances decision making, and stimulates adherence. Optimal nonverbal communication by the oncologist, particularly eye contact, body posture, and smiling, presumably benefits patients’ trust. We were the first to experimentally examine (1) how the oncologist’s nonverbal behavior influences trust, and (2) individual differences in breast cancer patients’ trust. Analogue patients (APs) viewed one out of eight versions of a video vignette displaying a consultation about chemotherapy treatment. All eight versions varied only in the oncologist’s amount of eye contact (consistent vs. inconsistent), body posture (forward leaning vs. varying), and smiling (occasional smiling vs. no smiling). Primary outcome was trust in the observed oncologist (Trust in Oncologist Scale). 214 APs participated. Consistent eye contact led to stronger trust (*β* = −.13, *p* = .04). This effect was largely explained by lower educated patients, for whom the effect of consistent eye contact was stronger than for higher educated patients (*β* = .18, *p* = .01). A forward leaning body posture did not influence trust, nor did smiling. However, if the oncologist smiled more, he was perceived as more friendly (*r*_s_ = .31, *p* < .001) and caring (*r*_s_ = .18, *p* = .01). Older (*β* = .17, *p* = .01) and lower educated APs (*β* = −.25, *p* < .001) were more trusting. Trust was weaker for more avoidantly attached APs (*β* = −.16, *p* = .03). We experimentally demonstrated the importance of maintaining consistent eye contact for breast cancer patients’ trust, especially among lower educated patients. These findings need to be translated into training for oncologists in how to optimize their nonverbal communication with breast cancer patients while simultaneously managing increased time pressure and computer use during the consultation.

**Electronic supplementary material:**

The online version of this article (doi:10.1007/s10549-015-3486-0) contains supplementary material, which is available to authorized users.

## Introduction

Trust in the oncologist is of paramount importance for breast cancer patients [1]. A breast cancer diagnosis comes with uncertain prospects, complex medical information and decisions, and impactful treatment. If cancer patients trust their oncologist in this vulnerable situation, they experience less worry [2, 3], improved decision making [4–6], and are more adherent to the oncologist’s recommendations [7–9]. Breast cancer patients are on average young and well-informed [10, 11]. Trust among cancer patients with these characteristics was found to be weaker than average [12]. Therefore, improving trust relations may particularly benefit patients with breast cancer.

Physicians’ verbal messages have been shown to influence patients’ interpersonal trust [13]. However, how physicians convey the message—their nonverbal communication—is likely as important as the information’s verbal content [14]. Nonverbal behavior is all communication produced by something other than words [15], and encompasses facial behavior, gaze, interpersonal distance, body movement, touch, vocal behaviors, and more [16]. Nonverbal communication substantially influences one’s perception of a conversation [14, 17, 18]. Consequently, it most likely has a crucial influence on trust [19].

Three nonverbal behaviors that are potentially influential for patients’ trust in their physician are eye contact, body posture and smiling. First, eye contact most saliently determines patients’ perceptions and evaluations of physicians [20]. Consistent eye contact between physicians and their patient is associated with an increase in patients’ satisfaction, disclosure and understanding [21–25]. At the same time, the mounting use of computers and electronic patient files during the consultation creates a challenge to physicians in maintaining consistent eye contact with their patient [26, 27]. Nowadays, the computer may even be regarded a ‘third person’ in the consultation room [28]. The more physicians gaze at their monitor, the less emotionally responsive they may become, causing patients to share less socio-emotional and psychosocial information [29]. Patients may perceive a physician who faces the screen instead of them as less attentive and available [30]. In this light, it is important to know what influence eye contact by the oncologist has on patients’ trust.

Second, oncologists’ body posture may influence trust. With their posture, physicians can convey their sense of involvement. If physicians keep a smaller physical distance (forward leaning) and a direct body orientation towards the patient, this appears to lead to more positive patient evaluations [31–33]. Oncologists’ body posture may, however, be affected by increased time pressure. A perception of restricted time could reduce the sense of involvement oncologists convey to patients. However, the evidence thus far is meager and the influence of oncologists’ body orientation on trust has not been investigated to date.

A third nonverbal behavior of particular relevance in oncology, is smiling and the use of humor between physician and patient. Both are not uncommon in the oncology setting, although intuitively, the ‘graveness’ of the oncology setting does not lend itself for lightheartedness. Nevertheless, a smile within a medical consultation may convey various desirable signals, such as encouragement, sympathy, or understanding [34]. In specific populations of elderly and immigrant cancer patients, occasional smiling by the oncologist appears to enhance trust and satisfaction [35, 36]. However, we do not know whether and how in general, smiling by the oncologist enhances or harms patients’ trust.

It should be noted that there is no ‘one size fits all’ approach to communication. Patients’ socio-demographic and personality characteristics may determine how they perceive nonverbal communication and, consequently, their trust. A personality trait consistently linked to trust is patients’ attachment style, i.e., how they relate to others in a dependent relationship [37]. People are able to form secure relationships to a higher (securely attached) or lower (insecurely attached) extent. Insecurely attached cancer patients appear to be less trusting of their oncologist [38, 39]. Moreover, they seem to perceive communication differently than securely attached patients [13].

Concluding, both nonverbal communication by the oncologist and characteristics of the patient could influence breast cancer patients’ trust in the oncologist. Most of these effects have, however, not been systematically studied. Moreover, all existing evidence for the importance of nonverbal communication is cross-sectional. Studies focusing on breast cancer patients are particularly rare. Therefore, we experimentally tested first, how breast cancer patients’ trust can be enhanced through the oncologist’s nonverbal communication, focusing on the effects of eye contact, body posture and smiling. Secondary outcomes were patients’ likelihood of recommending the oncologist to others and affective perception of the oncologist. Second, we tested how patient characteristics, i.e., age, education and attachment style, influenced trust directly and indirectly, through patients’ perception of nonverbal communication.

## Methods

### Design

To investigate effects of isolated nonverbal behaviors experimentally, we used scripted video vignettes, i.e., videotaped medical consultations based on scripts. This prevents practical and ethical issues that would arise from manipulating communication in clinical practice. Video vignettes have proven practical, feasible, and externally valid [13, 40–43].

A basic vignette was created first. Next, variations of this video were created, identical in content except for the nonverbal behaviors displayed by the oncologist. Combining these variations in a 2 × 2 × 2 design resulted in eight different video vignettes. Participants were randomized to video versions. Reported trust in the observed oncologist was the primary outcome. Secondary outcomes were patients’ likelihood of recommending the oncologist to others and affectve perception of the oncologist.

### Subjects and procedure

Women participated as analogue patients (APs), i.e., viewing the video while imagining themselves as the patient. The validity of the APs approach has been substantiated [40]. However, it is still unclear whether recruiting actual breast cancer patients as APs yields more externally valid results than involving healthy women. Therefore, we approached: (1) patients with a previous breast cancer diagnosis and (2) healthy women of comparable age. Patients were recruited through cancer patient organizations and radiotherapy out-patient clinics of a regional and an academic hospital. They were invited to apply through e-mail (patient organizations) or information letter (out-patient clinics). Healthy women were recruited through a snowballing method, via participating patients.

Applicants were further informed by phone and received an e-mail with a web link to the experiment. Online, APs first filled out a baseline survey assessing socio-demographic and medical background characteristics (T0). Next, they viewed a randomly selected variant of the video. APs were instructed to play the video full screen and sufficiently loud, and to make sure they were not interrupted during viewing. They were specifically instructed to imagine themselves being the patient in the video. After viewing, in a second survey, APs evaluated the observed oncologist (T1, primary and secondary outcomes).

### Development of experimental conditions

Video vignettes development is described in detail in Appendix A (ESM). A basic script was created first, describing a consultation between a medical oncologist and a breast cancer patient about adjuvant chemotherapy after a mastectomy. Whereas such consultations normally last between 15 and 60 min, we shortened our script to last no more than 10 min for practical reasons.

**Fig. 1:**
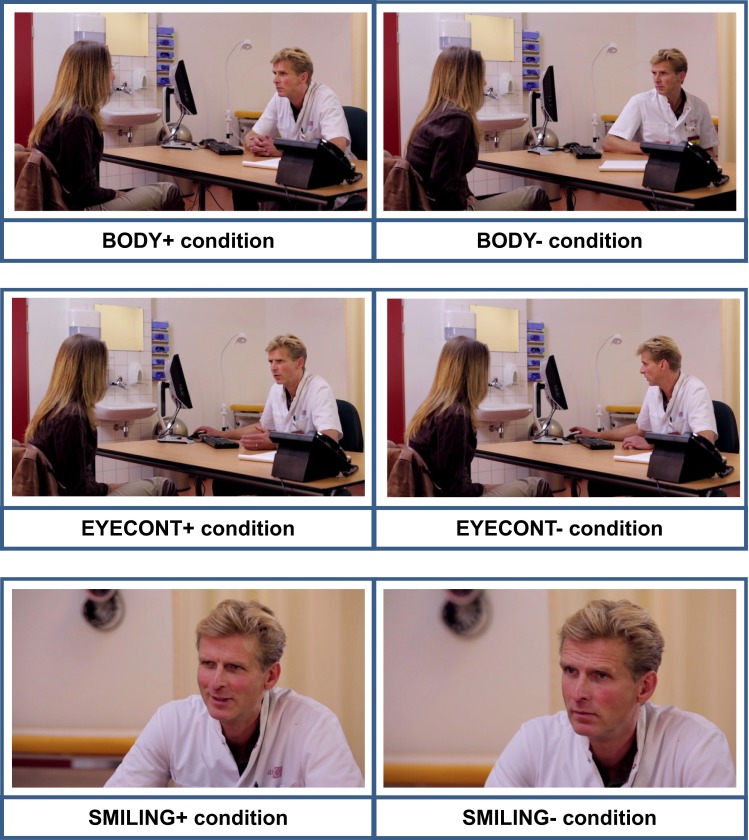
Illustration of the difference between nonverbal conditions

Variations of the basic script were created for the oncologist’s amount of eye contact (EYEC+: consistent vs. EYEC−: inconsistent), body posture (BODY+: frontal forward leaning, vs. BODY−: varying), and amount of smiling (SMILING+: occasional vs. SMILING−: never) (see Fig. 1; Box 1). Manipulations were based on previous literature on the effects of nonverbal communication, and on videotapes of radiotherapy consultations [44], in which the naturally occurring variation in nonverbal behaviors was assessed.

**Box 1:** Specification of nonverbal manipulations

(1) Eye contact (a) Consistent (EYECONT+): The oncologist retains the patient’s gaze throughout the patient’s speech and refrains from looking at the computer screen or paperwork while talking or listening (b) Inconsistent (EYECONT−): The oncologist frequently gazes at the computer screen or paperwork while providing information or when the patient speaks [24, 25, 29]
(2) Body posture (a) Forward leaning and frontal (BODY+): The oncologist is seated directly facing the patient, leaning slightly forward over the table (b) Varying (BODY−): The oncologist alternates between a forward leaning, patient-directed posture and a backward leaning posture, leaning away at a 45° angle from the patient [31, 33]. Gazing at the computer was intentionally unrelated to leaning away from the patient, to keep the two manipulations distinct
(3) Smiling (a) Occasional (SMILING+): the oncologist smiles occasionally, especially in the first and final phases of the consultation which involves more social talk. Smiles are modest, conveying understanding or encouragement [34] (b) Never (SMILING−): the oncologist does not smile throughout the consultation

Trained actors performed as the (male) oncologist and (female) patient. Pilot-testing was conducted, first on the basic script and second on test-fragments of the video vignettes. Pilot procedures, results and consequent adaptations to the vignettes are described in Appendix A (ESM). Figure 1 illustrates differences between the conditions using screenshots from the videos.

### Measures

#### Socio-demographic, medical, and personality characteristics (T0)

Age and education level were measured. Among patients, we assessed time since diagnosis, treatment status and number of contacts with their present oncology specialist. Attachment style was measured using the 12-item experiences in close relationships short form (ECR-sf) [39, 45]. The ECR-sf distinguishes two dimensions: *attachment anxiety* (6 items), a fear of rejection or abandonment and need for approval, and *attachment avoidance* (6 items), a fear of interpersonal intimacy and need for self-reliance [45]. An example of an item is ‘*I worry that my close ones won’t care about me as much as I care about them*’ (7-point Likert scale, *completely disagree* = 1 to *completely agree* = 7). Reliability was *α* = .73 for attachment avoidance and *α* = .76 for attachment anxiety.

#### Operationalization success (T1)

Each video version was scored on: (1) the percentage of time in which the oncologist maintained eye contact with the patient (2) the percentage of time in which the oncologist kept a forward leaning/frontal versus backward leaning/away body posture, and (3) the number of smiles, using behavioral observation software (The Observer [46]). In addition, participants rated their perception of the oncologist’s amount of eye contact, physical distance (to assess body posture), and smiling behavior (3 single items, 5-point Likert scale, *completely disagree* = 1 to *completely agree* = 5).

#### Realism and engagement (T1)

Three items measured how realistic, credible, and likely to happen in real life patients perceived the video-events (7-point Likert scale, *completely disagree* = 1 to *completely agree* = 7). Participants’ engagement in the video was assessed using the Video Engagement Scale (VES; 15 items, 7-point Likert scale: *completely disagree* = 1 to *completely agree* = 7; *manuscript submitted*).

#### Primary outcome: trust in the observed oncologist (T1)

Trust in the observed oncologist was assessed with the 18-item *Trust in Oncologist Scale* [47, 48]. This scale assesses trust using 18 items, answered on a five-point Likert scale (*completely disagree* = 1 to *completely agree* = 5). An example item is *‘This doctor strongly cares about your health’* (5-point Likert scale: *completely disagree* = 1 to *completely agree* = 5). Reliability of the scale was *α* = .95.

#### Secondary outcome measures (T1)

The secondary outcomes were participants’ (1) likelihood of recommending the oncologist to others and their (2) affective perception of the oncologist, i.e., his competence, friendliness, hurriedness, caring, and honesty. Both were measured with single items, on a five-point Likert scale.

### Statistical analysis

All analyses were conducted using SPSS 20.0 [49]. To test eight effects, i.e., three main effects of the manipulations, three main effects of patients’ characteristics (age, education, participant group), and two effects of personality characteristics (attachment anxiety, and attachment avoidance), using an alpha of .05, for a 95 % power to detect medium-sized effects (Cohen’s *F* ^2^ = .15), a minimum sample size of 160 was required [50].

Using stepwise regression analysis, we first tested the main effects of the nonverbal communication manipulations on trust. Second, APs’ background and personality characteristics were added. Third, all possible interactions between communication manipulations and AP characteristics were added to the regression. Regression analyses were repeated, replacing ‘trust’ with the secondary outcome variable ‘likelihood of recommending the oncologist to others’. For regression analyses, all variables were centralized around the mean (continuous variables) or scored as −0.5 versus 0.5 (dichotomous variables). The variable ‘education level’ was dichotomized as higher (college or university) or lower. Finally, we explored the correlation between nonverbal communication manipulations and APs’ affective perception of the oncologist. To account for multiple testing, findings were considered significant at *p* < .01 and marginally significant at *p* < .05.

## Results

### Sample

The sample included 214 participants—147 (69 %) patients and 67 (31 %) healthy women (Table 1). Because the questionnaire included forced responses, there were no missing values. Average age was 54 years (range 31–91). Mean trust in the observed oncologist was 3.30 (SD = 0.73, range 1.00–5.00). For attachment anxiety (ECR-sf), average score was 2.72 (SD = 1.15, range 1.00–6.83) and for attachment avoidance 2.58 (SD = 1.07, range 1.00–6.00).

**Table 1:** Demographic, health, and relationship characteristics of the sample (*N* = 214)

	Breast cancer patients (*n* = 147)		Healthy women (*n* = 67)	
	Median (range)	SD	Median (range)	SD
Age (*n* = 214)	55 (31–91)	11	51 (31–73)	11

	*N*	%	*N*	%
Educational level (*n* = 214)				
None/primary school	1	1	0	0
Secondary/lower level vocat. school	84	57	31	46
College/university	61	42	36	54
Current living situation (*n* = 214)				
Alone	30	20	6	9
With partner	63	43	21	31
With partner and children	48	33	33	49
Other	6	4	7	11
Self-reported treatment status (*n* = 147)				
In active treatment (incl. endocrine therapy)	58	39		
Undergoing regular check ups	86	59		
No treatment or check ups	3	2		

	Mean	SD		
Number of months since diagnosis (*n* = 139)	42	47		

### Manipulation check

Mean percentage of eye contact in the EYECONT+ conditions was 84 % (range 83–85 %), compared to 56 % (range 54–60 %) in the EYECONT− conditions. For the BODY+ conditions, forward leaning was observed in 92 % (range 90–94 %) of the consultation, compared to 62 % (range 60–64 %) in the BODY− conditions. In the SMILING+ conditions, there were on average 8 smiles (range 7–9), compared to 1 smile (range 0–2) in the SMILING− conditions. Participants perceived the eye contact and smiling manipulations as intended: the oncologist in the EYECONT+ was perceived as having more eye contact (*M* = 3.73, SD = 0.94) than in the EYECONT− condition (*M* = 3.18, SD = 1.08; *t* = −3.96, *p* < .001). The oncologist in the SMILING+ conditions was perceived as smiling more (*M* = 3.20, SD = 0.92) than in the SMILING− conditions (*M* = 2.26, SD = 0.88; *t* = −7.64, *p* < .001). The manipulation for body posture was not consciously perceived: perception of the physical distance between oncologist and patient was equal for the BODY+ (*M* = 3.32, SD = 1.06) and the BODY− (*M* = 3.42, SD = 1.05; *t* = 0.73, *n.s.*) conditions.

### The effects of nonverbal communication

Below, we report results for Step 2 in regression analysis, including all main effects of nonverbal communication and patient characteristics, on trust (Table 2) and ‘Likelihood of recommending the oncologist to others’ (Table 3). Consistent eye contact led to stronger trust (*β* = −.13, *p* = .04), as well as to a higher reported likelihood of recommending the oncologist to others (*β* = −.16, *p* = .02). Moreover, if the oncologist maintained consistent eye contact, he was perceived as more caring (*r*_s_ = .16, *p* = .02). Variation in body posture did not influence trust (*β* = −.08, *p* = .22), nor likelihood of recommending the oncologist to others (*β* = −.07, *p* = .33). However, forward leaning did cause patients to perceive the oncologist as more medically competent (*r*_s_ = .15, *p* = .03). Smiling by the oncologist did not enhance trust (*β* = −.08, *p* = .24) and only slightly increased the likelihood of recommending the oncologist to others (*β* = −.13, *p* = .05). If the oncologist smiled, he was perceived as more friendly (*r*_s_ = .31, *p* < .001) and caring (*r*_s_ = .18, *p* = .01).

**Table 2:** Main and interaction effects of socio-demographic characteristics, communication manipulations, and attachment on trust (*TiOS*) in multiple regression analysis

	*b*	SE *b*	*β*	*p*
Step 1				
Constant	0.00	.05		
Eye contact by oncologist	−0.20	0.10	−0.14	.05
Body posture of oncologist	−0.14	0.10	−0.10	.17
Smiling by oncologist	−0.14	0.10	−0.10	.16
Step 2				
Constant	0.02	0.05		
Eye contact by oncologist	−0.19	0.09	−0.13	.04
Body posture of oncologist	−0.11	0.09	−0.08	.22
Smiling by oncologist	−0.11	0.09	−0.08	.24
Age	0.01	0.01	0.17	.01
Education	0.36	0.10	0.25	<.001
Patient type (healthy vs. patient)	0.19	0.10	0.12	.07
Attachment avoidance	−.11	0.05	−0.16	.03
Attachment anxiety	0.08	0.05	0.12	.09
Step 3				
Constant	0.02	0.05		
Eye contact by oncologist	−0.15	0.09	−0.10	.12
Body posture of oncologist	−0.11	0.09	−0.08	.23
Smiling by oncologist	−0.15	0.09	−0.11	.11
Age	0.01	0.01	0.21	<.01
Education	0.38	0.10	0.26	<.001
Patient type (healthy vs. patient)	0.17	0.10	0.11	.10
Attachment avoidance	−0.12	0.05	−0.18	.02
Attachment anxiety	0.09	0.05	0.14	.05
Eye contact × age	0.01	0.01	0.05	.48
Eye contact × education	0.53	0.20	0.18	<.01
Eye contact × attach avoid	−0.06	0.10	−0.04	.59
Eye contact × attach anxi	0.02	0.09	0.17	.81
Body posture × age	0.01	0.01	0.06	.36
Body posture × education	0.29	0.20	0.10	.14
Body posture × attach avoid	−0.01	0.10	−0.01	.90
Body posture × attach anxi	0.04	0.09	0.03	.67
Smiling × age	−0.02	0.01	−0.12	.09
Smiling × education	−0.31	0.19	−0.11	.11
Smiling × attach avoid	0.10	0.10	0.07	.33
Smiling × attach anxi	−0.08	0.09	−0.06	.37

*R* ^2^ =  .04 for Step 1 (*p* < .05*), Δ*R* ^2^ = .14 for Step 2 (*p* < .001***), Δ*R* ^2^ =  .07 for Step 3 (*p* = .11)

**Table 3:** Main effects of socio-demographic characteristics, communication manipulations, and attachment on ‘likelihood of recommending the oncologist to others’ in multiple regression analysis

	*b*	SE *b*	*β*	*P*
Step 1				
Constant	0.00	.07		
Eye contact by oncologist	−0.32	0.13	−0.16	.02
Body posture of oncologist	−0.13	0.13	−0.07	.33
Smiling by oncologist	−0.27	0.13	−0.14	.04
Step 2				
Constant	0.02	0.06		
Eye contact by oncologist	−0.31	0.13	−0.16	.02
Body posture of oncologist	−0.11	0.13	−0.06	.40
Smiling by oncologist	−0.25	0.13	−0.13	.05
Age	0.01	0.01	0.14	.05
Education	−0.40	0.13	0.20	<.01
Patient type (healthy vs. patient)	0.06	0.14	0.03	.69
Attachment avoidance	−0.09	0.07	−0.10	.18
Attachment anxiety	0.09	0.06	0.11	.14
Step 3				
Constant	0.25	0.07		
Eye contact by oncologist	−0.26	0.13	−0.14	.04
Body posture of oncologist	−0.13	0.13	−0.07	.33
Smiling by oncologist	−0.29	0.13	−0.15	.03
Age	0.01	0.01	0.15	.03
Education	0.37	0.14	0.19	<.01
Patient type (healthy vs. patient)	0.05	0.14	0.02	.72
Attachment avoidance	−0.10	0.07	−0.11	.16
Attachment anxiety	0.11	0.06	0.13	.08
Eye contact × age	0.01	0.01	0.04	.56
Eye contact × education	0.55	0.27	0.14	.04
Eye contact × attach avoid	0.17	0.14	0.09	.22
Eye contact × attach anxi	−0.15	0.13	−0.09	.425
Body posture × age	0.01	0.01	0.05	.46
Body posture × education	0.16	0.27	0.04	.56
Body posture × attach avoid	−0.01	0.14	−0.01	.95
Body posture × attach anxi	0.07	0.13	0.04	.56
Smiling × age	−0.02	0.01	−0.11	.14
Smiling × education	−0.29	.27	−0.08	.28
Smiling × attach avoid	0.23	0.14	0.12	.10
Smiling × attach anxi	−0.18	.13	−0.11	.15

*R* ^2^ = .05 for Step 1 (*p* < .05*), Δ*R* ^2^ = .08 for Step 2 (*p* < .01**), Δ*R* ^2^ = .06 for Step 3 (*p* = .24)

### Patients’ socio-demographic characteristics and attachment style as predictors

Age as well as education level predicted the level of trust in the observed oncologist: older (*β* = .17, *p* = .01) and lower educated APs (*β* = −.25, *p* < .001) were more trusting. Older (*β* = .14, *p* = .05) and lower educated APs were also more likely to recommend the oncologist to others (*β* = .20, *p* < .01).

Higher attachment avoidance predicted lower trust in the observed oncologist (*β* = −.16, *p* = .03), but was not related to the likelihood of recommending the oncologist to others (*β* = −.10, *p* = .18). Higher attachment anxiety did not relate to trust (*β* = .12, *p* = .09), nor likelihood of recommending the oncologist (*β* = .11, *p* = .14). In Step 3, only one of the twelve interactions between patient characteristics and nonverbal communication on trust was significant: the effect of consistent eye contact on trust was only present for APs with a lower education level (*β* = .18, *p* = .01). Inspection of the interaction effect revealed that for highly educated APs (college or university), the effect of eye contact was virtually absent. As a result of adding this interaction effect in Step 3 of regression analysis, the main effect of eye contact on trust became non-significant (*β* = −.10, *p* = .12).

## Discussion

This is the first experimental study testing the impact of nonverbal communication behavior by the oncologist on breast cancer patients’ trust. Results indicate that maintaining eye contact by the oncologist enhanced trust, particularly in the lower educated. A forward leaning and frontal body posture did not significantly improve trust, although it did lead to the oncologist being perceived as more competent. Smiling by the oncologist did not lead to stronger trust. Yet, an oncologist who smiled was perceived as more friendly and caring, and evoked a higher willingness in patients to recommend him to others.

The observed influence of eye contact on trust confirms its importance for rapport building [51]. We experimentally demonstrated this effect, which was previously found only in cross-sectional studies. The extent of eye contact was manipulated independently of other verbal and nonverbal behaviors, ensuring one effect could not be ascribed to other factors. Lower educated breast cancer patients appeared to benefit specifically from consistent eye contact by the oncologist, whereas highly educated patients did not. These results provide useful suggestions to oncologists on how to improve their nonverbal communication, especially with lower educated patients, who are overall more vulnerable. Improved nonverbal communication will contribute to mutual trust.

Maintaining frequent eye contact is hampered by the increasingly dominant role of the computer and use of the electronic health record in the consultation room. In a recent study, a substantial subset of physicians using a health electronic record gazed at the computer during as much as 50 % of the consultation [27]. Physicians who spend less time gazing at the computer have more active interactions with their patient [52] and are more patient-centered [53]. Teaching oncologists to manage computer tasks while simultaneously keeping in contact with patients is essential for maintaining good treatment relations. This could mean developing strategies to maintain the flow of conversation while using the computer and preventing periods of extended gaze at the computer and prolonged silence [53]. On the other hand, the advantages of the computer can be maximized, for example, by actively sharing information with patient [54].

Body posture did not influence trust as strongly as expected. This could lead to the conclusion that the oncologist’s posture does not contribute to patients’ trust. Alternatively, our manipulation may not have been sufficiently strong, as it did not influence viewers’ perception of the physical distance kept by the physician. More consistent or drastic changes in the physician’s posture might influence patients’ perception of the oncologist as distant or hurried and, consequently, impair their trust. In previous research, for example, a standing oncologist was perceived as much less compassionate and calm than one who sat [55, 56]. Standing behavior even affected patients’ perception of time: a sitting physician was perceived as spending more time with the patient, even when the consultation was shorter than that of an oncologist who stood [57]. In most Dutch oncology out-patient settings, the oncologist is seated behind a table opposite the patient. The presence of that table allows for less variation in the oncologist’s body posture. Nevertheless, physical nearness and attention, expressed by leaning forward over the table and orienting towards the patient, have been consistently linked to patients’ positive perceptions of physicians. Thus, future research should shed more light on how physicians can convey an optimal sense of involvement through their posture behind the table and how this relates to trust.

Smiling did not lead to enhanced trust in the present study. Nevertheless, patients did perceive the smiling oncologist as more friendly and caring. Possibly, smiling only influenced the ‘caring’ dimension of trust, but not other aspects, such as perception of honesty and medical competence [1]. Alternatively, smiling affects trust for a subgroup of patients [36, 58]. In line with this hypothesis, the observed influence of smiling on trust in this study was stronger for lower educated patients. Possibly, higher educated patients pay more attention to cognitive than to affective aspects of communication.

In addition to nonverbal communication, patients’ characteristics predicted breast cancer patients’ trust. First, older patients were found to report stronger trust, as in previous studies [59–61]. Second, education predicted trust as well as the likelihood of recommending the oncologist to others. Previous studies provided conflicting evidence about the relation between education level and trust [13, 59, 60]. In the present study, lower educated patients reported significantly stronger trust. Possibly, lowly educated patients take a less critical stance in their relation with the oncologist than those more highly educated. Third, more avoidantly attached patients reported weaker trust in the oncologist, in accordance with previous results [38, 39] and with the well-established difficulty people with avoidant attachment styles have to form trusting relationships [38, 62].

All of these individual differences indicate that for certain subgroups of patients, trust is less evident than for others. For oncologists, it is important to realize that younger, higher educated breast cancer patients and patients who are avoidantly attached are less inclined to fully trust them, at least initially. Efforts should be directed at identifying which communication strategies enhance trust among these specific subgroups. For example, to meet avoidantly attached patients’ needs, oncologists may emphasize patients’ autonomy and independence [62–64]. Younger and higher educated patients may be more likely to consult alternative information sources like the internet, or seek complementary and alternative treatment, which may reduce their trust in the oncologist [65, 66]. Inquiring about patients’ information seeking behavior may raise oncologists’ awareness of (the content of) alternative information viewed by the patient. For lower educated patients, efforts can be made to prevent them from blindly and without question obeying the oncologist. Subsequently, oncologists can optimally tailor their communication to patients’ background and personality.

A limitation of our study should be acknowledged. The advantages of using an experimental design—i.e., studying behaviors in isolation—also form a disadvantage: in reality, nonverbal behaviors often occur in combinations. Moreover, we could only manipulate three nonverbal behaviors, whereas many other may also be of relevance (paralinguistics, facial expressivity, hand gestures). In the present study, we manipulated only the behaviors most frequently linked to trust. In future studies, other meaningful nonverbal behaviors and combinations of behaviors could be studied.

## Conclusion

This study showed that both nonverbal communication by the oncologist, particularly maintaining eye contact, influences breast cancer patients’ trust. These findings need to be translated into training for oncologists focused on maintaining contact nonverbally with breast cancer patients while simultaneously managing the computer and increased time pressure. By clarifying which nonverbal communication strategies can be taught and how [67], we can enable evidence-based recommendations for clinical practice. Combined with the earlier established contribution of verbal communication, this study has enabled the further understanding of breast cancer patients’ trust in their oncologist.

## Electronic supplementary material

Below is the link to the electronic supplementary material.

Supplementary material 1 (PDF 391 kb)
