# Supplementary material for: All eyes on the patient: the influence of oncologists’ nonverbal communication on breast cancer patients’ trust
Source: Breast Cancer Res Treat. 2015 Jul 31;153(1):161–71. doi: 10.1007/s10549-015-3486-0 (PMC4536267; doi:10.1007/s10549-015-3486-0)
Supplement: Supplementary file 1 — Supplementary material 1 (PDF 391 kb) [file 10549_2015_3486_MOESM1_ESM.pdf]

Appendix A to manuscript
"All eyes on the patient – the influence of oncologists' nonverbal communication on breast cancer patients' trust"

Video Vignettes Development

Marij A. Hillen[1*], Hanneke C.J.M. de Haes[1], Geertjan van Tienhoven[2], Nina Bijker[2], Hanneke W.M. van Laarhoven[3], Daniëlle M. Vermeulen[1], Ellen M.A. Smets[1]

1. Department of Medical Psychology - Academic Medical Center, University of Amsterdam, P.O. Box 22700, 1100 DE Amsterdam, The Netherlands.
2. Department of Radiotherapy - Academic Medical Center, University of Amsterdam, P.O. Box 22700, 1100 DE Amsterdam, The Netherlands.
3. Department of Medical Oncology - Academic Medical Center, University of Amsterdam, P.O. Box 22700, 1100 DE Amsterdam, The Netherlands.

*Corresponding author. E-mail: M.A.Hillen@amc.uva.nl. Tel: +31.20.566.4631. Fax: +31.20.566.9104

Creation of the basic script

The basic script was based on audiotaped and videotaped consultations about adjuvant chemotherapy for breast cancer (n=6). Whereas such consultations normally last between 15 and 60 minutes, we shortened our script to last no more than 10 minutes. This was because scripting a longer consultation would be practically difficult, and would moreover make our manipulations less impactful. The scripted consultation involves a 49-year-old female breast cancer patient and a 44-year old male medical oncologist, meeting for the first time after the patient has undergone conservative breast surgery. The oncologist summarizes the patient's history, explains the tumor characteristics, associated risks and prognosis (using a computer programme) and proposes a treatment plan involving chemotherapy as well as hormonal therapy. Subsequently, he discusses the chemotherapy procedure, side effects and risks. He does not discuss hormonal therapy in detail. At the end of the consultation, the patient is provided the opportunity to ask questions.

Validation, Stage 1

To assess validity of the basic script, two medical oncologists, four researchers of medical communication, one movie director and 3 patients with breast cancer commented upon the script's credibility, fluidity, accurateness of medical content and realism. The most important subsequent changes made to the script were:

1. The consultation was more explicitly introduced at the start, to help viewers' engagement in the story
2. The questions asked by the patient were kept more simple
3. More pauses were explicitly written in the script, to slow down the pace of the consultation

Development of manipulations

Apart from the manipulations, the scripts were kept identical across conditions. We selected three behaviors described in the literature on nonverbal communication in medical settings, which have previously been most frequently linked to trust in the physician. Nonverbal variations in the basic script were created for the oncologist's (1) amount of eye contact, (2) body posture, and (3) amount of smiling. Operationalizations were based on previous literature on the effects of nonverbal communication, and on observation of earlier videotaped recordings of introductory radiotherapy consultations [1], in which we assessed the naturally occurring variation in nonverbal behaviors within a Dutch radiotherapy setting. These consultations strongly resemble the introductory consultation in a medical oncology setting, with regard to structure and duration. We used behavioral coding software, i.e., The Observer [2], to time-stamp behavioral events within the recordings of radiotherapy consultations. For eye contact, we documented the percentage of the consult in which eye contact between physician and patient took place. For body posture, we assessed variation in and duration (in seconds) of body postures, specifically forward/backward leaning and orientation. For smiling, we assessed the range in frequency of smiles. Next, we constructed the two levels of our manipulation according to the maximum and minimum within the range of the observed behaviors.

1) Eye contact
    a. Consistent eye contact (EYECONT+): The oncologist retains the patient's gaze throughout the patient's speech and refrains from looking at the computer screen or paperwork while talking or listening.
    b. Inconsistent eye contact (EYECONT-): The oncologist frequently gazes at the computer screen or paperwork while providing information or when the patient speaks [3-5].

2) Body posture
   a. Forward leaning and frontal (BODY+): The oncologist is seated directly facing the patient, leaning slightly forward over the table.
   b. Varying posture (BODY-): The oncologist alternates between a forward-leaning , patient-directed posture and a backward leaning posture, leaning away at a 45° angle from the patient [6, 7]. Gazing at the computer was intentionally unrelated to leaning away from the patient, to keep the two manipulations distinct.

3) Smiling
   a. Occasional smiling (SMILING+): the oncologist smiles occasionally, especially in the first and final phases of the consultation which involves more social talk. Smiles are modest, conveying understanding or encouragement [8].
   b. No smiling (SMILING-): the oncologist does not smile throughout the consultation

We combined the manipulations in every possible way, resulting in 8 video versions (2 x 2 x 2) video versions. Thus, an example of a video version is EYECONT-, BODY+, SMILING+. Care was taken to manipulate nonverbal behaviors independently of each other.

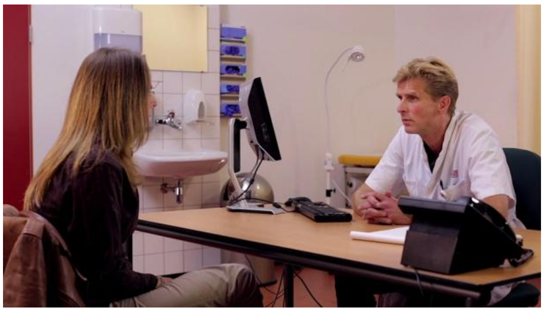
BODY+ condition

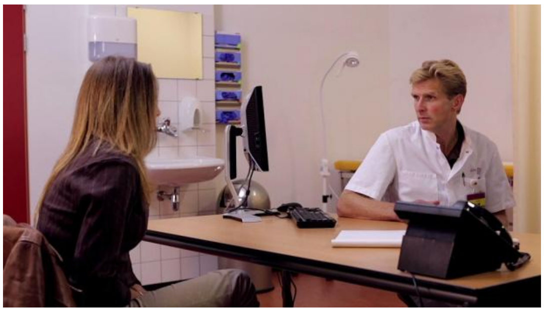
BODY- condition

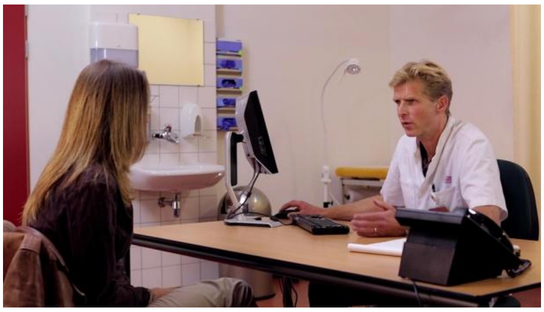
EYECONT+ condition

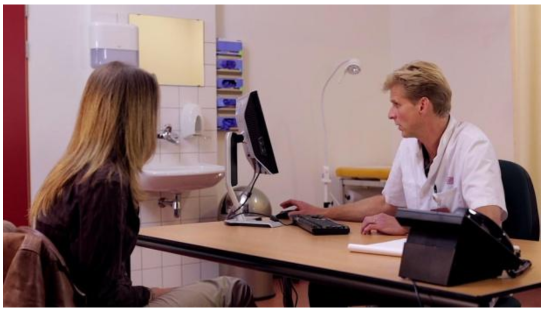
EYECONT- condition

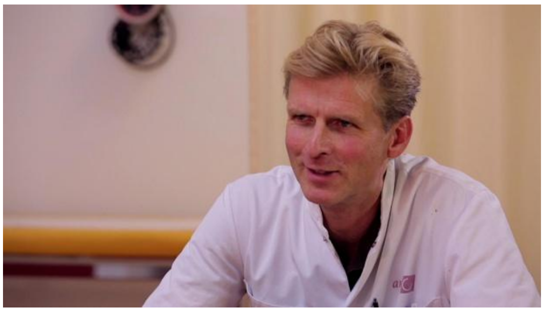
SMILING+ condition

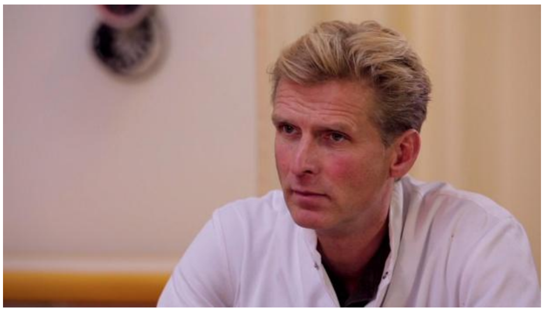
SMILING- condition

Recording of the scripts to video

Trained actors acted as the (male) oncologist and (female) patient. Actors were chosen instead of a real patient and oncologist, since the experimental nature of the study would require them to adhere strictly to the script. Moreover, the oncologist would need to repeatedly perform the script, while specifically varying aspects of his nonverbal behaviour and keeping all other communication constant. Recordings were made in a large outpatient medical consultation room. The oncologist sat behind a desk wearing a white coat. To enhance viewers' engagement, the patient's point of view was employed throughout most of the recording. This means that the camera was directed towards the oncologist while looking over the patient's shoulder. Only three times, the camera faced the patient for one or two seconds. Two cameras were used for the patients' point of view, one with a zoom shot and one with a 'medium wide' shot.

Validation, Stage II

For pilot-testing, segments of the scripts were role-played by the actors at the end of a training session. We recorded two verbally identical segments, lasting approximately one minute each, in which nonverbal communication varied, resulting in 6 fragments in total. Each pilot participant viewed four video-fragments; both variations of two out of three nonverbal manipulations. Two medical oncologists, six researchers of medical communication, two healthy women and four former breast cancer patients participated. Both internal validity (manipulation success) and external validity (realism) were assessed.

Internal validity

To test whether manipulations were perceived as intended, four questions were used for each nonverbal behavior, to be answered on a 10-point Likert scale. For bodily posture, participants rated their perception of the oncologist's 1) physical distance from the patient, 2) involvement with the patient, 3) sense of calm, and 4) professionalism. For eye contact, participants assessed the oncologist's 1) amount of eye contact with the patient, 2) involvement, 3) interest, and 4) attention. For smiling, participants rated the oncologist's 1) amount of smiling, 2) kindness, 3) comforting attitude, and 4) seriousness. The items were analyzed separately as well as combined for each nonverbal behavior (i.e., by averaging scores on the four items).

Table 1 indicates that the manipulations for eye contact were successful: the condition with more eye contact was perceived as such. Although the means for smiling and forward-leaning body posture were both higher compared to the no smiling and backward-leaning body posture conditions, these differences were not significant. When looking at the individual items however, participants did perceive a small difference in the amount of smiling. The oncologist in the SMILING- video was perceived to smile less ($M = 4.60$, $SD = 1.51$) than in the SMILING+ video ($M = 6.40$, $SD = 2.61$; $t = -2.45$, $p = .07$). No difference was found on individual items between the two conditions for body posture. The oncologist in the BODY- condition was perceived to have an equal physical distance to the patient ($M = 5.11$, $SD = 1.45$) as in the BODY+ condition ($M = 4.78$, $SD = 2.33$; $t = .329$, $p = .751$).

Based on these results, manipulations of body posture and smiling were enhanced. In the BODY+ condition, the oncologist was instructed to keep both elbows on the table at all times while leaning forward. In the BODY- condition, the oncologist sat backward with only his fingers on the table, and at times turned his body away from the patient, in a 45° angle. Individual remarks by pilot

participants indicated that the smiles by the oncologist were at times somewhat unnatural, or looked nervous. The actor was therefore instructed to convey more comforting and encouraging smiles.

Table 1 T-test contrasting average scores on four items, to assess manipulation success

	N	M	(SD)	t*	p
**Body posture**					
BODY-	**9**	6.389	.936		
BODY+	**9**	6.750	1.581	-.495	.634
**Eye contact**					
EYECONT-	**7**	4.429	1.742		
EYECONT+	**8**	7.156	1.18	-3.130	.020
**Smiling**					
SMILING-	**5**	5.75	1.311		
SMILING+	**5**	6.55	1.745	-1.835	.140

*paired samples t-test

External validity

To assess realism, participants answered two questions (‘*how realistic were the events in this video?*’ and ‘*how believable were the events in this video?*’; 10-point Likert scale). Scores on the two items were averaged. Pilot participants (N = 12) rated realism with a mean of 7.48 (SD 1.25). Answers on additional open-ended questions suggested that the communication between patient and oncologist could improve on authenticity, e.g., by slowing down the pace of the conversation. This was communicated to and practiced by the actors for the final videos.

All pilot participants except for the doctors were asked how much they could identify with the patient in the video (10-point Likert scale). Participants (n = 10) scored a mean of 6.29 (SD 2.58). Answers on additional open-ended questions on how to increase the ability to identify with the patient suggested that the actor who played the patient at times came across as unnatural in her response to the oncologist. Changes were made by allowing the patient more time to think and process, and to change her tone of voice while speaking about emotional subjects. Based on additional remarks, small changes were made to the set up of the desk by adding a note pad, and to the script if the conversation did not flow naturally. Moreover, extra attention was paid to maintaining continuity in the videos while recording different variations.

Referenties

1. Douma KFL, Koning cce, Zandbelt LC et al. Do patients' information needs decrease over the course of radiotherapy? Supportive Care in Cancer 2012; 20: 2167-2176.

2. Noldus LPJJ. The Observer: a software system for collection and analysis of observational data. Behavior Research Methods, Instruments, & Computers 1991; 23: 415-429.
3. Hall JA, Harrigan JA, Rosenthal R. Nonverbal behavior in clinician patient interaction. Appl Prev Med 1995; 4: 21-37.
4. Griffith CH, Wilson JF, Langer S, Haist SA. House staff nonverbal communication skills and standardized patient satisfaction. J Gen Intern Med 2003; 18: 170-174.
5. Margalit RS, Roter D, Dunevant MA et al. Electronic medical record use and physician-patient communication: An observational study of Israeli primary care encounters. Pat Educ Couns 2006; 61: 134-141.
6. Weinberger M, Greene JY, Mamlin JJ. The Impact of Clinical Encounter Events on Patient and Physician Satisfaction. Social Science & Medicine Part E-Medical Psychology 1981; 15: 239-244.
7. Larsen KM, Smith CK. Assessment of Nonverbal-Communication in the Patient-Physician Interview. Journal of Family Practice 1981; 12: 481-488.
8. Henry SG, Fuhrel-Forbis A, Rogers MAM, Eggly S. Association between nonverbal communication during clinical interactions and outcomes: A systematic review and meta-analysis. Pat Educ Couns 2012; 86: 297-315.
